# Questionnaire-based survey on the distribution and incidence of canine babesiosis in countries of Western Europe

**DOI:** 10.1051/parasite/2014015

**Published:** 2014-03-14

**Authors:** Lénaïg Halos, Isabelle Lebert, David Abrial, Fabien Danlois, Karin Garzik, Daniel Rodes, Monika Schillmeier, Christian Ducrot, Jacques Guillot

**Affiliations:** 1 Merial 29 avenue Tony Garnier 69007 Lyon France; 2 INRA, UR346 63122 Saint-Genès-Champanelle France; 3 Merial Benelux Leonardo Da Vincilaan 19 1831 Diegem Belgium; 4 Merial GmbH Am Söldnermoos 6 85399 Hallbergmoos Germany; 5 BIPAR, UMR ENVA, ANSES, UPEC, Dynamyc, École Nationale Vétérinaire d’Alfort, UPE 94704 Maisons-Alfort France

**Keywords:** *Babesia* spp., Canine babesiosis, Incidence, Geographic distribution, Europe

## Abstract

The incidence of canine babesiosis may vary considerably from one country to another depending on the distribution of the causative parasite species and their specific vectors. The aim of the present study was to evaluate the clinical occurrence of canine babesiosis diagnosed in European veterinary clinics and propose an updated map of the disease distribution in Western Europe. Questionnaires were sent to companion animal veterinary clinics in Spain, France, Benelux, Germany and Austria. The annual number of babesiosis cases in 2010, the number of practitioners in the clinic and the location of the clinic were recorded. The total numbers of dogs and practitioners in each country were used for definition of the reference populations and the annual incidence of canine babesiosis was calculated by dividing the total number of reported babesiosis cases by the total number of dogs in the veterinary practices involved in the study. Data were georeferenced for distribution map construction. The overall annual incidence of clinical babesiosis amongst the investigated dog population was 0.7%, with significant variations amongst countries and regions. Three epidemiological situations were described: (i) Spain, with co-existence of several species of piroplasms and patchy distribution of babesiosis, (ii) France, with overall presence of babesiosis due to *Babesia canis* and local variations and (iii) Benelux, Germany and Austria, with overall low prevalence of the disease associated with localised description related either to imported cases or to small autochthonous foci of *B. canis* infection.

## Abstract (fr)

L’incidence de la babésiose canine varie considérablement d’un pays à l’autre en fonction de la distribution des espèces de parasites en cause et de leurs vecteurs. Le but de cette étude était d’évaluer l’expression clinique de la babésiose canine diagnostiquée dans les cliniques vétérinaires européennes et de proposer une carte actualisée de la distribution de la maladie en Europe de l’Ouest. Des questionnaires ont été envoyés aux cliniques vétérinaires canines en Espagne, en France, au Benelux, en Allemagne et en Autriche. Le nombre annuel de cas de babésiose en 2010, le nombre de praticiens de la clinique et l’emplacement de la clinique ont été recueillis. Le nombre total de chiens et de praticiens dans chaque pays a été utilisé pour définir les populations de référence et l’incidence annuelle de la babésiose canine a été calculée en divisant le nombre total de cas de babésiose signalés par le nombre total de chiens dans les cliniques vétérinaires de l’étude. Les données ont été géo-référencées pour construire la carte de distribution. L’incidence annuelle globale de la babésiose clinique dans la population de chiens étudiée était de 0,7 % avec d’importantes variations entre les pays et les régions. Trois situations épidémiologiques ont été décrites : (i) l’Espagne avec la coexistence de plusieurs espèces de piroplasmes et une distribution inégale de la babésiose, (ii) la France avec une présence généralisée de la babésiose due à *Babesia canis* et des variations locales, (iii) le Benelux, l’Allemagne et l’Autriche avec une faible prévalence générale de la maladie associée à des descriptions localisées soit de cas importés ou de petits foyers autochtones d’infection par *B. canis.*

## Introduction

Babesiosis (or piroplasmosis) is a worldwide emerging tick-borne haemoprotozoosis affecting many mammalian species. Pathogenesis is related to intraerythrocytic multiplication of apicomplexan parasites of the genera *Babesia* and *Theileria*, also called piroplasms, which are some of the most ubiquitous and widespread blood parasites in the world [10]. Babesiosis is characterised by erythrocyte destruction, causing mild to severe systemic clinical manifestations. Piroplasms are transmitted by hard ticks and are capable of infecting a wide variety of vertebrate hosts with high host specificity [10]. The geographical distribution of the causative agents and thus the occurrence of babesiosis in animals are largely dependent on the habitat of relevant vector tick species.

The first cases of canine babesiosis were described in Europe in 1895 [19] and since, the spectrum of *Babesia* pathogens that infect dogs is gradually being elucidated, especially with the aid of molecular techniques and clinical investigations. To date, around eight different species have been shown to be responsible for babesioses in dogs worldwide [20].

In Europe, four species of *Babesia* or *Theileria* infecting dogs have been reported and may co-exist in some areas [7, 15, 20]. Two of them are large *Babesia* described as endemic for decades. *Babesia canis* is transmitted by *Dermacentor reticulatus* and distributed, as its tick vector, in temperate cold climates of Western Europe and Eastern Europe, whereas *Babesia vogeli* is transmitted by *Rhipicephalus sanguineus* and located in the Mediterranean area (southeast of France, central and southern Italy, Spain, Portugal and Greece). In addition, two species of small *Babesia* have been reported recently. *Babesia gibsoni* is sporadically reported in Europe as a consequence of the introduction of infected dogs from abroad (mainly from Asia, the US or Australia). A newly described *Babesia microti*-like species, also called *Theileria annae,* is endemic in northern Spain where it causes severe infection in dogs [6, 7]. For these two latter species, the tick vector identification remains uncertain: *R. sanguineus* is probably involved in *B. gibsoni* transmission, whereas *Ixodes hexagonus* is suspected to transmit *T. annae* [4].

The virulence of these different species in dogs is variable. Dogs infected by *B. canis* typically present with the acute form of babesiosis, which is characterised by the association of a febrile syndrome and a haemolytic syndrome and may be life-threatening; the species *B. vogeli* is generally thought to be less virulent, causing subclinical, mild or moderate clinical signs in adult dogs [3, 12, 20]. Infection by *T. annae* and *B. gibsoni* piroplasms also causes severe illness [7]. Microscopy is reasonably sensitive for detecting large intraerythrocytic *Babesia* in stained blood smears from capillary beds [3, 12]. Moreover, babesiosis rarely resolves spontaneously in the absence of specific treatment. Large forms of *Babesia* are commonly treated with imidocarb dipropionate with good clinical response, allowing a therapeutic diagnosis [3, 18, 20].

The occurrence of the disease in Europe is variable and dependent on the vector distribution. There is limited data on the incidence of the diagnosis of the disease by veterinarians in the field. Whilst France is known to be endemic for babesiosis due to *B. canis,* with hyperendemic areas in the southwestern part of the country [3, 8, 18], the disease was considered as exotic in Benelux and Germany until recent years. In the last few decades, outbreaks of autochthonous babesiosis, caused by *B. canis*, have occurred in the Netherlands [16], in the Saarland in Germany [2] and in Norway [17], underlining the spread of the infection over Europe. In Spain, several diseases co-occur according to the localisation: in northern Spain the occurrence of babesiosis due to *B. canis* is described, whereas *B. vogeli* is reported in the southern part of the country [20]. The northwestern part of the country is considered as a hyperendemic area for *T. annae* [4, 7]. Despite several reports and clinical case descriptions, no large-scale surveys have been conducted in Europe to assess the impact of the disease amongst the general dog population.

The objective of the present study was to evaluate the incidence of clinical occurrence of canine babesiosis diagnosed in veterinary clinics in seven countries of Western Europe: Spain, France, Belgium, Luxembourg, the Netherlands, Germany and Austria. The study was based on a questionnaire survey sent to veterinary clinics to report the number of new clinical cases of babesiosis diagnosed in 2010.

## Material and methods

### Definition of cases and questionnaire

The cases were defined as clinical cases of babesiosis of dogs diagnosed by the veterinarian practitioners in their day-to-day practice over the course of a year (2010). Standardised questionnaires (Appendix 1) were sent between June and September 2011 to the majority of companion animal veterinary clinics in Spain, France, Benelux, Germany and Austria. Questionnaires were sent either by email, or distributed by hand and filed at the clinic. The total number of questions was limited in order to encourage participation: the postcode of the clinic, the number of cases diagnosed in the clinic during the whole year (2010) and the number of veterinarians treating companion animals at the clinic.

### Estimate of the dog population

In order to calculate the incidence of the disease, i.e., the percentage of the dog population that contracted babesiosis during the period of the study, reference data about veterinarian and dog populations were required. As it was not possible to obtain the annual number of dogs presented in each clinic, it was estimated based on the number of companion animal veterinarians in the clinic multiplied by the average dog population per companion animal veterinarian in the country; the latter was calculated as the total number of dogs in the country divided by the number of companion animal veterinarians in the country. These data were collected as follows.

The number of companion animal veterinarians per country was obtained thanks to the data collected by the different veterinary national associations or by specific report studies: the Bundestierärztekammer (BTK, Berlin) in Germany, Syndicat National des Vétérinaires d’Exercice Libéral (SNVEL, Paris) in France, Union Professionnelle Vétérinaire (UPV, Nivelles) in Belgium and Luxembourg, the Dutch veterinary association (KNMvD, Houten) in the Netherlands and data collected for the European project Leonardo Da Vinci II [14] in Spain. As those databases comprised data of veterinarians whose practice is totally devoted to companion animals and data of veterinarians whose practice is shared between companion and production animals, the number of companion animal practitioners as used in the present study was the sum of the total number of companion animal veterinarians and half the number of veterinarians of mixed practice in France, Germany and the Netherlands. In Belgium, Luxembourg, Austria and Spain, only data from companion animal veterinarians were used as the number of veterinarians sharing their activity between companion and production animals was considered negligible.

There is no official figure on canine populations available in the countries of the European Union. The most accurate estimates are those obtained from the European Pet Food Industry Federation (available online: http://www.fediaf.org/facts-figures/).

As a summary, the mean number of dogs per companion animal veterinarian in each country (data not shown) was calculated as follows:

$$\mathrm{mean}\;{\mathrm{DOG}}_{\mathrm{vet}-\mathrm{country}}=\;{\mathrm{DOG}}_{\mathrm{country}}/{\mathrm{VET}}_{\mathrm{country}},$$

where DOG_country_ is the number of dogs per country and VET_country_ is the number of companion animal veterinarians per country.

$${\mathrm{VET}}_{\mathrm{country}}=\;{\mathrm{VET}}_{\mathrm{companion}}+0.5\;\times {\mathrm{VET}}_{\mathrm{mixed}},$$

where VET_companion_ is the number of companion animal veterinarians per country and VET_mixed_ is the number of veterinarians of mixed practice per country.

### Computation of the annual incidence

Annual Canine Babesiosis (CB) incidence in a given area was defined as the number of new cases occurring during the one-year period in this area. The annual canine babesiosis incidence (CB Inc_region_) was calculated for each area by dividing the total number of newly affected dogs in all the clinics of the area that answered the questionnaire by the estimated dog population in these clinics following the formula:

$$\mathrm{CB}\;{\mathrm{Inc}}_{\mathrm{region}}=\;\sum_{\mathrm{region}}^{}\mathrm{CB}\times 100/\left(\left(\sum_{\mathrm{region}}\mathrm{CAV}\right)\times \mathrm{mean}\;{\mathrm{DOG}}_{\mathrm{vet}-\mathrm{country}}\right),$$

where CB corresponds to the cases of canine babesiosis registered during the study, CAV corresponds to the participating companion animal veterinarians and mean DOG_vet-country_ corresponds to the mean number of dogs per companion animal veterinarian per country as described above.

The annual incidence was computed at regional and European levels. The score statistic [1] was used to calculate 95% confidence intervals and to compare the annual incidence of each region with the European incidence considered as the reference. No score test was performed when the number of babesiosis cases was less than five. Regions were categorised into three categories according to the difference from the reference: no difference, significantly lower and significantly higher than the reference.

### Map building

The georeferencing of 82 administrative regions of Spain, France, Belgium, Luxembourg, Germany, the Netherlands and Austria was performed with ESRI Data & Maps (2005, Redlands, USA). The annual canine babesiosis incidence per region was spatially referenced to region numbers and mapped using Quantum GIS Software (version 1.8.0). All regions with an annual incidence significantly different from the annual overall incidence at the European level were specifically mentioned on the map (score test).

## Results

### Questionnaire response level and raw data collection

Responses were received from clinics representing 3674 veterinarians and covering 71 out of 82 regions of the seven countries. The total number of babesiosis cases registered in 2010 by the veterinarians who answered the questionnaire ranged from 0 in Luxembourg to 12,064 in France. The reference data, response level and number of babesiosis cases registered by the participating veterinary clinics are shown in Table 1.

**Table 1.** Reference data on dogs and veterinarians per country used for the analyses and data (number of answers and number of cases of canine babesiosis) obtained in the survey.

	Austria	Belgium	France	Germany	Luxembourg	The Netherlands	Spain
Total number of administrative regions with answers/Total number of administrative regions	6/9	3/3	22/22	15/16	1/1	9/12	15/19
Total number of dogs in the country*	61,2000	1,330,700	7,595,000	5,300,000	35,000	1,493,000	4,720,000
Total number of vets in the country**	1000	2370	10,652	10,437	45	3608	8500
Number of vets included in the study	151	462	1884	570	4	274	329
*Response level (%)*	*15.1*	*19.5*	*17.7*	*5.5*	*8.9*	*7.6*	*3.9*
Number of canine babesiosis cases diagnosed in 2010	163	38	12,064	150	0	13	2383

* Data from the European Pet Food Industry Federation (http://www.fediaf.org/facts-figures/).

** Data from the Bundestierärztekammer (BTK, Berlin) in Germany, Syndicat National des Vétérinaires d’Exercice Libéral (SNVEL, Paris) in France, Union Professionnelle Vétérinaire (UPV, Nivelles) in Belgium and Luxembourg, the Dutch veterinary association (KNMvD, Houten) in the Netherlands, Leonardo Da Vinci II in Spain.

### Annual incidences and geographic distribution

**Figure 1.**
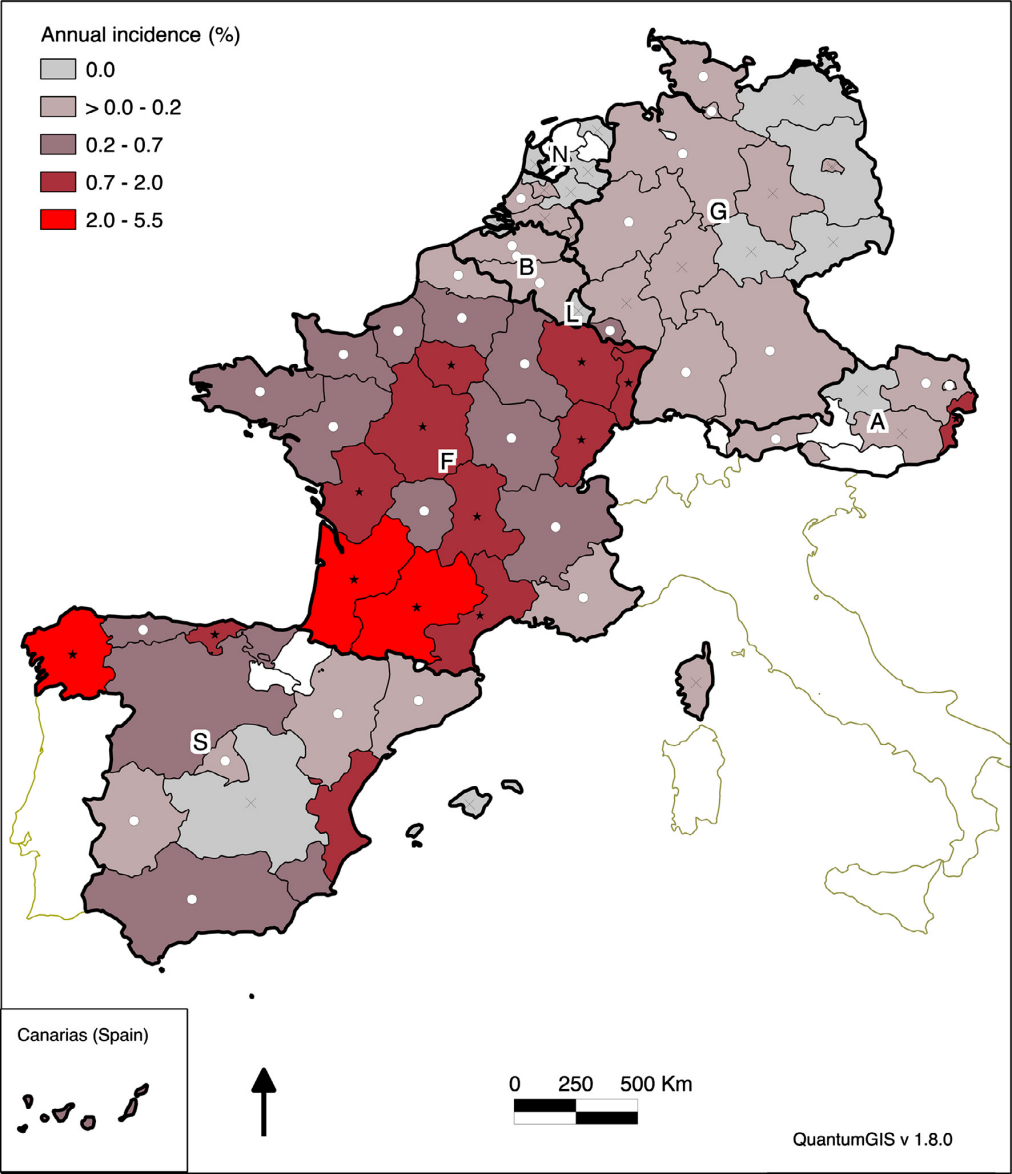
Geographical distribution of the incidence of canine babesiosis amongst the general dog population in Western Europe regions in 2010. Initials of the countries are indicated as: Spain (S), France (F), Belgium (B), Luxembourg (L), the Netherlands (N), Germany (G) and Austria (A). The colour gradient (from grey to red) indicates an increasing incidence. Score tests compared the regional incidence and the Western European incidence. The white circle indicates that regional incidence was significantly lower than the Western European incidence. The star indicates that regional incidence was significantly higher than the Western European incidence. The cross indicates that no score test was applicable.

The overall annual incidence of clinical babesiosis amongst the dog population in Western Europe was 0.70% (95% confidence interval (CI95) 0.69–0.71%) with large variations amongst the countries, and amongst the regions in each country (0%–5.5%). The distribution of incidence calculated across Western Europe is reported in Figure 1. Spain displayed a heterogeneous pattern including three hyperendemic foci: one in northwestern Spain (Galicia) (5.5%), one in northern Spain (Cantabria) (1.6%) and another on the southeastern Mediterranean coast (0.9%) (Valencia). Babesiosis in France is widely distributed, with seven regions with an incidence above 1.0%: three main foci were located in southwestern France, reaching the highest incidence (2.4%), continuously linked to a central core including Massif Central (1.1%) and Ile de France (0.9%). The overall incidence in northern Europe including Benelux, Germany and Austria was low. In Germany, cases were reported mainly from the southwestern part of the country. The Saarland, at the French border, displayed the highest incidence (0.3%). In Austria, 110 out of 163 cases were reported from the eastern part of the country (Burgenland, 0.9%). In Benelux, no region appeared as hyperendemic. In Belgium, 22 out of 38 cases were diagnosed in an area of 30 km around Mons in Wallonia. In the Netherlands, all 13 cases were reported from the southwestern part of the country.

## Discussion

This study offers the first large-scale picture of the clinical incidence of babesiosis in Western Europe. The collected results are in line with what has been reported in previous local studies [3, 4, 7, 11, 16, 20] and allow us to map the disease as diagnosed in the field by veterinarian practitioners across Western Europe. Whilst the accuracy has to be considered in regard to the response levels and the country coverage, the results of the present study clearly illustrate the heterogenic distribution of the disease across Western Europe.

Practitioner questionnaire surveys provide a highly informative approach to estimating the incidence of animal diseases and contribute to a better understanding of the evolution of the diseases over large geographic areas [5]. The major biases of questionnaire-based epidemiological surveys are the potential inaccuracies linked to failures in diagnosis (over- or underestimation), limited response levels or reliability of the reference population estimates. These biases should be considered in the present study, even though efforts were made to reduce their potential impact. The figures for reference populations were estimated based on the most accurate databases available on canine populations and veterinary distribution in order to offer a reliable analysis.

Underestimation of incidence could result from cases that were not presented to the veterinary surgeon or from diagnostic failure. The latter situation may principally occur in the case of chronically infected dogs or animals with atypical or subclinical presentations. Overestimation might be due to false diagnosis by the veterinarian practitioners. Differential diagnoses include a number of causes of anaemia, such as other vector-borne diseases, especially in Spain and southern France, where *Ehrlichia canis* and *Hepatozoon canis* are also present [7, 20, 21]. In northern countries, vector-borne diseases are less frequent and do not usually present with a haemolytic syndrome (for example, Lyme borreliosis). Generally, babesiosis due to *B. canis* presents with acute clinical signs [12]; however, the therapeutic success of imidocarb is a good indicator for a field diagnosis of babesiosis and diagnoses conducted on the basis of clinicopathological findings have a high accuracy level (93.5%) [13]. This study thus gives a good estimate of the incidence of acute babesiosis in the field.

The response level was variable from one country to another and may have limited the accuracy of the results from countries where it was under 10% (Spain, Germany, Benelux, the Netherlands). Nevertheless, the distribution of the disease across Western Europe resulting from this survey reflects what is known from local studies and gives a large-scale overview of the activity of the disease in Western Europe. The very variable pattern of the disease from one country to another, and inside a country from one region to another, is clearly observable.

The situation in Spain is complex because the different species of *Babesia/Theileria* and tick vectors co-exist across the country and Spain displayed the highest incidence disparity. *R. sanguineus*, which is the vector of *B. vogeli,* the less virulent parasite, is present in the south of the country, whereas *D. reticulatus* is located on the North Atlantic coast as well as *I. hexagonus,* the suspected vector of *T. annae*. The present study clearly identified three hyperendemic foci, which correspond to the previously described areas of distribution of each species [20]. Galicia, known to be the endemic area for *T. annae,* appears as a hyperendemic place for the clinical expression of babesiosis in the field. This very high incidence may be related to the co-existence in this area of *T. annae* and *B. canis*, both pathogenic species. These results are to be taken with caution due to the limited response level obtained in Spain. Further studies on a smaller scale in the endemic region of Spain would offer a more accurate understanding of the pathogenic system in this multi-parasite and multi-vector country.

In France, the response level and country coverage were high and both the number of companion animal veterinarians and the number of dogs at a “department” (smaller administrative unit) level were available. As a consequence, babesiosis distribution in France was described at a departmental level in a specific study [8]. Canine babesiosis in France is mainly due to *B. canis* transmitted by *D. reticulatus*. Interestingly, *R. sanguineus*, the vector of *B. vogeli*, and *D. reticulatus* tick species co-exist in southwestern France, where prevalence is the highest. *B. vogeli* may contribute to increasing the prevalence of the disease [18].

The overall incidence in Northwestern Europe is low, with some localised occurrences in hotspot areas known to be potentially endemic for *D. reticulatus*. Some sporadic cases observed in areas where the disease is not present were related to imported pathogens in dogs with a history of travel in Southern Europe. In Germany, Saarland was identified as endemic for canine babesiosis. This feature has been previously described and *D. reticulatus* populations have been reported in that region [2]. In Austria, the concentration of cases at the Hungarian border is not surprising as the disease is widespread in Hungary [9, 11]. More than 50% of the 38 cases in Belgium were reported in an area of 30 km around Mons in Wallonia. This area is known as endemic for canine babesiosis due to *B. canis* (E. Claerebout, *personal communication*). Amongst other cases, a travel history to France and Spain was reported for two dogs. Such is the case in the Netherlands where 10/13 of the infected dogs had a travel history in southern Europe, especially in France. As the number of questions in the questionnaire was limited, no more information is available on the history of these probably imported cases.

In conclusion, three epidemiological situations may be described in Western Europe: (i) Spain (and maybe southern France), where several species of *Babesia* co-exist and where a patchy distribution of babesiosis is observed; (ii) France, where babesiosis (due to *B. canis*) is present almost everywhere with local variations; and (iii) Benelux, Germany and Austria, with overall low prevalence associated with localised reports of the disease related either to imported cases or to very limited foci of infection (by *B. canis*).

Further studies should be conducted in the different hyperendemic areas identified in the present study to understand the circulation of the disease amongst western European dog populations better. Babesiosis is a disease known to be highly dependent on specific biotopes with very heterogeneous distribution on a very small scale, correlated with a favourable environment for each tick vector species [3]. As a consequence, those further studies should include the characterisation of the tick populations.

## Competing interests

The authors declare that they have no competing interests.

## Appendix 1

Supplementary pdf file provided by the authors.
