# Supplementary material for: Questionnaire-based survey on the distribution and incidence of canine babesiosis in countries of Western Europe
Source: Parasite. 2014 Mar 14;21:13. doi: 10.1051/parasite/2014015 (PMC3952654; doi:10.1051/parasite/2014015)
Supplement: Supplementary file 1 — Supplementary pdf file provided by the authors. [file parasite-21-13-s1.pdf]

Questionnaire Canine Babesiose Western Europe

1) Date ____

2) Address of the clinic

____

____

2) Number of veterinarians working in the clinic

____

3)

**Number of cases of canine babesiosis diagnosed in the clinic last year (2010)**	

4) Additional remarks

